# Complete mitogenome sequence of *Rasbora argyrotaenia* (Actinopterygii: Cyprinidae)

**DOI:** 10.1080/23802359.2017.1347835

**Published:** 2017-07-07

**Authors:** Wahyu Endra Kusuma, Pratama Deffi Samuel, Dewa Gede Raka Wiadnya, Anik Martinah Hariati, Yoshinori Kumazawa

**Affiliations:** a Department of Aquaculture, Faculty of Fisheries and Marine Science, University of Brawijaya, Malang, Indonesia;; b Department of Aquatic Science, Faculty of Fisheries and Marine Science, University of Brawijaya, Malang, Indonesia;; c Department of Fisheries, Faculty of Fisheries and Marine Science, University of Brawijaya, Malang, Indonesia;; d Department of Information and Basic Science and Research Center for Biological Diversity, Graduate School of Natural Sciences, Nagoya City University, Nagoya, Japan

**Keywords:** *Rasbora*, East Java, phylogenetic tree

## Abstract

The mitochondrial genome of a small freshwater fish *Rasbora argyrotaenia* from Java Island, Indonesia, was completely sequenced. This mitochondrial genome had 16,740 bp in length and consisted of 37 genes in the typical vertebrate mitochondrial gene arrangement. Phylogenetic analysis showed that *R. argyrotaenia* is more closely related to *R. borapetensis* than to other Javanese rasboras, *R. aprotaenia* and *R. lateristriata*.

The silver rasbora *Rasbora argyrotaenia* is one of 66 *Rasbora* species naturally distributed in Indonesia. Among them, three rasboras occur in Java Island of Indonesia: *R. argyrotaenia*, *R. aprotaenia* and *R. lateristriata* (Kottelat 2013; Froese and Pauly 2017). Whereas complete mitogenomic sequences of *R. aprotaenia* and *R. lateristriata* have been determined (Kusuma and Kumazawa 2016), those of *R. argyrotaenia* have not been obtained.

A sample of *R. argyrotaenia* was collected in a tributary of Brantas River, the second longest river in Java Island, Indonesia (geographic coordinate: S 07°27′38.8″, E 112°28′46.2″). A small portion of right pectoral fin was excised and preserved in the TNESU8 buffer (Asahida et al. 1996) for subsequent DNA extraction, long PCR amplification of the mitogenome, and amplification, sequencing and assembly of shorter (650–950 bp) DNA fragments (Kusuma and Kumazawa 2016). The whole body specimen was preserved in ethanol and registered to the Specimen Depository, Faculty of Fisheries and Marine Science, University of Brawijaya under the voucher number UB.1.122.4.

The complete mitogenomic sequence of *R. argyrotaenia* thus determined (16,740 bp; DDBJ/EMBL/GenBank accession number LC269105) had 37 genes for 13 proteins, 22 tRNAs and 2 rRNAs together with a major non-coding region in a typical gene arrangement of vertebrate mitogenomes (Anderson et al. 1981). All protein genes had an ATG start codon except for the cytochrome oxidase subunit I gene with GTG as an initiation codon. Seven protein genes were terminated with TAA stop codon, whereas the remaining six genes required polyadenylation for the creation of stop codons in mRNAs. All tRNA genes can be folded into the standard cloverleaf secondary structures (Kumazawa and Nishida 1993).

A phylogenetic analysis (Figure 1) showed with a strong bootstrap probability (99%) that *R. argyrotaenia* is more closely related to *R. borapetensis* than to any other rasboras including *R. aprotaenia* and *R. lateristriata*. This is in agreement with a recent molecular phylogeny reconstructed using two mitochondrial and two nuclear gene sequences (Kusuma et al. 2016), supporting a view of at least two rounds of colonization of Java Island by rasboras in the past.

**Figure 1.**
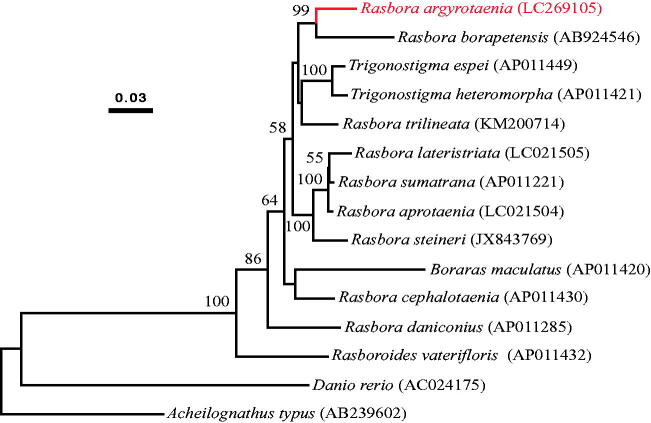
A maximum-likelihood tree illustrating the phylogenetic position of *R. argyrotaenia* among other *Rasbora* species. The maximum-likelihood analysis was conducted using concatenated amino acid sequences of 13 mitochondrial protein genes (3806 sites) and Garli v2.0 (Zwickl 2017) under the mtREV + IG substitution model. Numbers at each node are bootstrap probabilities by 500 replications shown only when they are 50% or larger. DDBJ/EMBL/GenBank accession numbers of mitogenomic sequences for each taxon are shown in parentheses.
